# Crystal structures of 4,4′-(disulfane-1,2-diyl)bis(5-methyl-2*H*-1,3-dithiol-2-one) and 4,4′-(diselanane-1,2-diyl)bis(5-methyl-2*H*-1,3-dithiol-2-one)

**DOI:** 10.1107/S2056989018007454

**Published:** 2018-05-22

**Authors:** Ivan Trentin, Claudia Schindler, Carola Schulzke

**Affiliations:** a Ernst-Moritz-Arndt-Universität Greifswald; Institut für Biochemie, Felix-Hausdorff-Strasse 4, 17487 Greifswald, Germany

**Keywords:** crystal structure, catenation, 1,3-ene-di­thiol-2-ones, di­sulfide, diselenide

## Abstract

The two title compounds, C_8_H_6_O_2_S_6_ and C_8_H_6_O_2_S_4_Se_2_, are isotypic with very similar cell parameters. The complete mol­ecules constitute the asymmetric units, despite being chemically perfectly symmetric. The most prominant differences in the metrical parameters arise from the distinct sizes of sulfur and selenium in the dichalcogenide bridges, with C—S—S—C and C—Se—Se—C torsion angles of 70.70 (5) and 68.88 (3)°, respectively. The crystal packing is determined by weak non-classical hydrogen-bonding inter­actions. One carbonyl oxygen but not the other participates in C—H⋯O inter­actions zigzagging along the *b* axis, forming infinite chains. This is complemented by an intra­molecular C—H⋯S inter­action and further inter­molecular C—H⋯S (C—H⋯Se) inter­actions, resulting in a three-dimensional network. The inter­actions involving the bridging chalcogenides form chains protruding along the *c* axis.

## Abstract 2

By *in situ* oxidation (S—S)^2−^ and (Se—Se)^2−^ moieties are formed, replacing ^*n*^Bu_3_Sn substituents on alkene carbon atoms of two distinct and subsequently linked 1,3-ene-di­thiol-2-one units. The resulting compounds, bis­[4-methyl-1,3-di­thiol-2-one] di­sulfide, C_8_H_6_O_2_S_4_S_2_, and bis­[4-methyl-1,3-di­thiol-2-one] diselenide, C_8_H_6_O_2_Se_6_, are isotypic.

## Chemical context   

Selenium- and sulfur-containing compounds play an important role in nature. Sulfur-rich compounds, in particular derivatives of tetra­thia­fulvalene and di­thiol­ene, comprise chemically inter­esting compounds with exceptional electronic structural characteristics. Selenium is an essential trace element in the active sites of several enzymes and plays *inter alia* an important role in anti­oxidant seleno­proteins for protection against oxidative stress such as in thio­redoxin reductase (Lee *et al.*, 1999 ▸; Lescure *et al.*, 1999 ▸; Mustacich & Powis, 2000 ▸; Watabe *et al.*, 1999 ▸; Williams *et al.*, 2000 ▸). In the di­sulfide isomerase protein family, thio­redoxin-like domains are rich in cysteine residues. A diselenide from seleno­cysteins was shown to be structurally very similar to the respective di­sulfide from two cysteins (Görbitz *et al.*, 2015 ▸). As a consequence, di­sulfide and diselenide compounds were developed as catalysts for oxidative protein folding and refolding reactions (Arai *et al.*, 2018 ▸). Here we report the serendipitous synthesis and structural characterization of bis­[3-methyl-1,3-ene-di­thiol-2-one] di­sulfide and bis­[3-methyl-1,3-ene-di­thiol-2-one] diselenide *via* unprecedented routes. Instead of the targeted products, the applied order of reactions yielded the novel di­sulfide and its diselenide analogue, which have potential applications in redox chemistry and as biologically inter­esting compounds. By *in situ* oxidation, S—S or Se—Se moieties are formed, replac­ing the ^*n*^Bu_3_Sn substituents of alkene carbon atoms of two distinct and consequently linked 1,3-ene-di­thiol-2-one units. As this constitutes a substitution of a ^*n*^Bu_3_Sn functional group, it is quite likely that this method can be applied to a variety of respective different precursors.
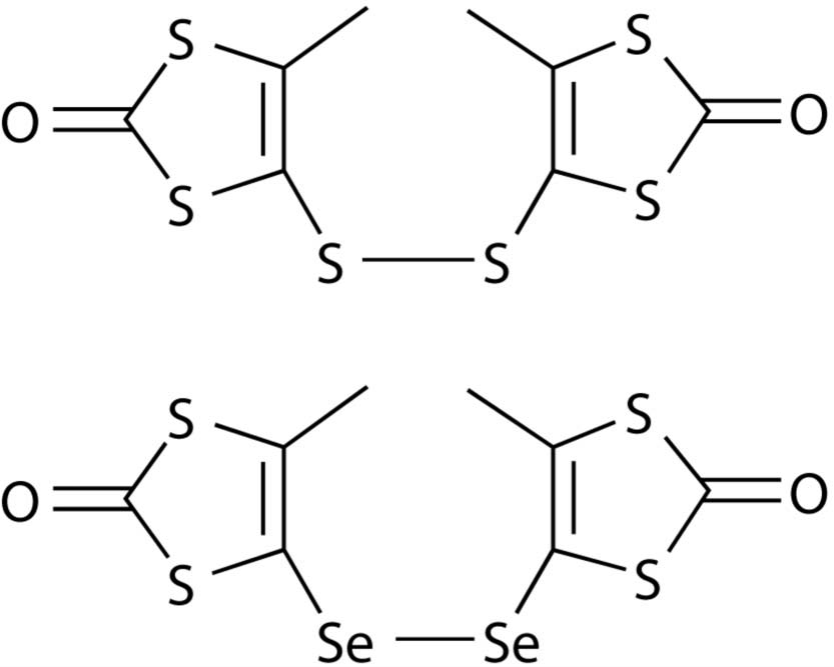


## Structural commentary   

**Figure 1:**
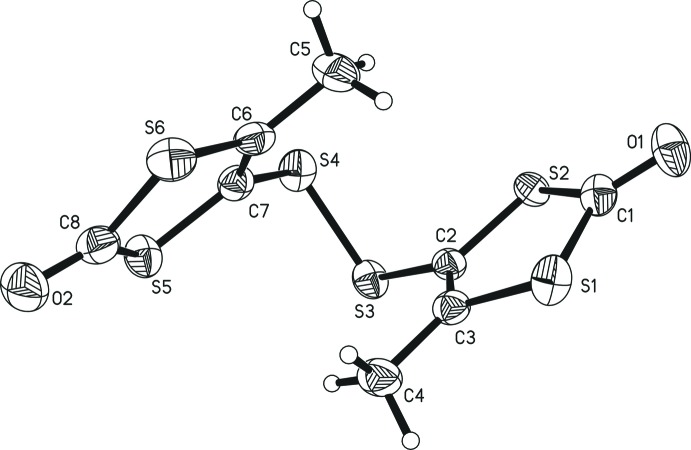
The mol­ecular structure of bis­[4-methyl-1,3-ene-di­thiol-2-one] di­sulfide. Displacement ellipsoids are shown at the 50% probability level.

The two title compounds are isotypic. One complete mol­ecule constitutes the asymmetric unit despite being chemically perfectly symmetric: *i.e*. no symmetry operation is used to generate the whole mol­ecular structure. In both compounds, two 3-methyl-1,3-ene-di­thiol-2-one moieties are linked by a dichalcogenide bridge (S_2_^2−^ or Se_2_^2−^), which is attached to one of the ene carbon atoms, while the other ene carbon is bound to a methyl group (Figs. 1 ▸ and 2 ▸). Both structures constitute the first examples of crystallographically characterized di­sulfides and diselenides in which two 1,3-ene-di­thiol-2-one moieties are linked by a dichalcogenide bridge. While related bridged 1,3-ene-di­thiol-2-thione moieties are reported for di­sulfides and also one compound in which the di­sulfide is part of a heterocycle with the 1,3-ene-di­thiol-2-one moiety (Chou *et al.*, 1998 ▸), no such analogues are known in the case of the diselenide bridge.

**Figure 3:**
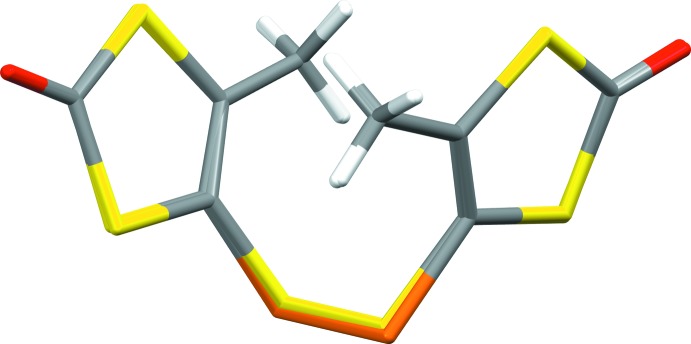
An overlay (*Mercury*; Macrae *et al.*, 2006 ▸) of the mol­ecular structures of bis­[4-methyl-1,3-ene-di­thiol-2-one] di­sulfide (yellow bridge) and bis­[4-methyl-1,3-ene-di­thiol-2-one] diselenide (orange bridge). The root-mean-square deviation (r.m.s.d.) and the maximum distance between atom positions are 0.078 and 0.171 Å, respectively.

The metrical parameters of both mol­ecules are nearly identical (see Fig. 3 ▸ for an overlay of the mol­ecules), with the largest differences found for the dichalcogenide bridge itself. The Se—Se distance [2.3397 (7) Å] is longer by *ca* 0.27 Å than the S—S distance [2.0723 (7) Å], matching almost exactly the difference in the respective covalent radii (0.13 Å; Pyykkö & Atsumi, 2009 ▸) multiplied by two. Similarly, the average C—Se distance [1.897 (4) Å] is longer by 0.15 Å than the average C—S distance [1.749 (2) Å]. Unusual electronic effects upon exchanging selenium for sulfur can, hence, be excluded. The average C—Se—Se angle [98.8 (6)°] is slightly more acute than the C—S—S angle [101.8 (6)°], which necessarily results from the longer distances involving the Se atom and the nearly identical atom positions of the 1,3-ene-di­thiol-2-thione moieties. All other differences in the metrical parameters between the two mol­ecular structures are marginal. All observed distances and angles also fall into or close to the expected/previously reported ranges. The S—S distances of the most closely related compounds range from 2.078 Å in an Fe(CO)_2_Cp-coordinating species (Matsubayashi *et al.*, 2002 ▸) to 2.160 Å in the [C_6_S_10_]^2−^ dianion crystallized as an ammonium salt (Breitzer *et al.*, 2001 ▸). The observed S—S distance [S3—S4; 2.0723 (7) Å] here is slightly shorter than the former, though not shorter than the lower limit of *ca* 2.00 Å when generally evaluating C—S—S—C linkages (Comerlato *et al.*, 2010 ▸; Aida & Nagata, 1986 ▸). Se—Se distances in compounds in which one Se_2_^2−^ unit binds to alkene carbon atoms and bridges two identical ene-moieties range from 2.303 Å (Biswas *et al.*, 2017 ▸) to 2.389 Å (Ruban *et al.*, 1981 ▸), with the Se—Se distance observed here [2.3397 (7) Å] falling right in the center of this range.

The structurally most notable features are the C—S—S—C and C—Se—Se—C torsion angles [70.70 (5) and 68.86 (3)°, respectively] which bring the two 1,3-ene-di­thiol-2-thione moieties in rather close proximity. In related di­sulfides they range from 52.08 to 109.82° (Breitzer *et al.*, 2001 ▸). C—S—S—C torsion angles near 90° were found *in silico* to stabilize structures by an overlap of one σ*S—C orbital with the 3*p* lone pair of the other sulfur atom, which is maximized in such an arrangement (Aida & Nagata, 1986 ▸). The observed C—Se—Se—C torsion angles of diselenide-bridged alkenes as the closest relatives of the title diselenide range from 73.03° (Ruban *et al.*, 1981 ▸) to 92.04° (Biswas *et al.*, 2017 ▸). In the crystalline solid state, apparently packing effects, steric bulk, hydrogen-bonding inter­actions, and π–π-stacking can influence the relative orientations of the two substituents on the di­sulfide unit significantly, whereas the values for alkene bridging diselenides observed to date are less varied.

The four 1,3-ene-di­thiol-2-one moieties (two in each structure) are essentially planar, with maximum deviations from the least-squares plane of 0.028 and 0.022 Å for the di­sulfide and for the diselenide, respectively, corresponding to the distances from atom S1 to the O1—S1—S2—C1—C2—C3 plane in both cases. The dihedral angles between the O1—S1—S2—C1—C2—C3 and the O2—S5—S6—C6—C7—C8 planes are 33.8 (2)° for the di­sulfide and 28.89 (11)° for the diselenide. Here, a smaller torsion angle around the dichalcogenide bridge is accompanied by a smaller angle between the two planes of the 1,3-ene-di­thiol-2-one moieties.

## Supra­molecular features   

**Figure 4:**
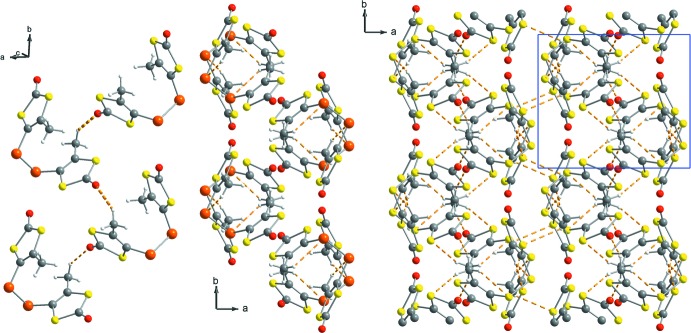
Packing and non-classical hydrogen-bonding motifs for the crystal structures of bis­[4-methyl-1,3-ene-di­thiol-2-one] di­sulfide and bis­[4-methyl-1,3-ene-di­thiol-2-one] diselenide. Left: C4—H4*B*⋯O1^i^ inter­actions zigzagging along the *b* axis shown for the diselenide; center: hydrogen-bonding inter­actions of the diselenide bridge C5—H5*C*⋯Se1^ii^ protruding along the *c* axis; right: additional symmetric hydrogen-bonding inter­actions between coplanar 1,3-ene-di­thiol-2-one moieties connecting adjacent chains shown for the di­sulfide (C4—H4*C*⋯S2^iv^ and C5—H5*C*⋯S5^iii^). For symmetry codes, see Tables 1 ▸ and 2 ▸.

In the crystals, mol­ecules are linked by C—H⋯O, C—H⋯S, and C—H⋯Se non-classical hydrogen-bonding inter­actions, some of which being comparably weak (Tables 1 ▸ and 2 ▸). One carbonyl oxygen but not the other participates in C4—H4*B*⋯O1^i^ inter­actions zigzagging along the *b* axis, forming infinite chains (Fig. 4 ▸, left). The respective *D⋯A* distances are 3.345 (2) Å for the disufide and 3.369 (5) Å for the diselenide. This is complemented by two intra­molecular inter­actions between the two chalcogens of the dichalcogenide bridges and the adjacent methyl substituents (C4—H4*A*⋯S3/Se1 and C5—H5*A*⋯S4/Se2) with *D⋯A* distances of 3.244 (2) for S3, of 3.234 (2) for S4, of 3.354 (4) for Se1, and of 3.341 (4) for Se2. Further inter­molecular C—H⋯S and C—H⋯Se inter­actions contribute to the formation of a three-dimensional network. The inter­actions involving the bridging chalcogenides form chains protruding along the *c* axis (Fig. 4 ▸, center and right). The closest 3-methyl-1,3-ene-di­thiol-2-one moieties of two adjacent mol­ecules are perfectly coplanar with the carbonyl oxygen atoms pointing into opposite directions. The respective distances between the planes are 3.55 and 3.58 Å for pairs of S1—S2—C1—C2—C3 heterocycles for the di­sulfide and diselenide, and 3.64 and 3.66 Å for pairs of S5—S6—C6—C7—C8 heterocycles. This arrangement fosters weak symmetric bidirectional C5—H5*C*⋯S5^iii^ and C4—H4*C*⋯S2^iv^ hydrogen-bonding inter­actions between methyl hydrogen atoms and S2 and S5 ring atoms, connecting adjacent chains and forming a three-dimensional network (Fig. 4 ▸, right).

## Database survey   

In the literature to date, only S—S-bridged 1,3-ene-di­thiol-2-*thione* compounds have been reported but no analogous 1,3-ene-di­thiol-2-*one* compounds (excluding those in which the ‘link’ is part of a heterocycle). The first such thione crystal structure was reported in 1999 by Cerrada *et al.*, which comprises an S—S-linked [C_3_S_5_—C_3_S_5_]^2−^ dianion (Cerrada *et al.*, 1999 ▸). Ten years later, Cerrada *et al.* described the S—S coupling *via* di­thiol­ate transfer from tin to nickel complexes where they isolated an S—S-bridged 1,3-di­thiol-2-thione with different substituents as a crystalline byproduct (Cerrada *et al.*, 2009 ▸). Rauchfuss and co-workers described the isolation and structural characterization of an S—S-linked dianion [C_6_S_10_]^2−^ as the tetra­methyl­ammonium salt (Breitzer *et al.*, 2001 ▸). In 2002, Matsubayashi *et al.* reported the formation of an S—S-linked [C_3_S_5_—C_3_S_5_]^2−^ system bridging two Fe(CO)_2_Cp complexes by coordination of thiol­ate sulfur to iron (Matsubayashi *et al.*, 2002 ▸). Wardell and coworkers carried out the controlled oxidation of cesium 4-benzoyl­thio-1,3-di­thiole-2-thione-5-thiol­ate using iodine as oxidant and obtained bis­(4-benzoyl­thio-1,3-di­thiole-2-thione)-5,5-di­sulfide, in two polymorphic forms (Comerlato *et al.*, 2010 ▸). Recently the formation of a di­sulfide with a 4-(methyl­sulfan­yl)-2*H*-1,3-di­thiole-2-thione unit was reported from the reaction of a Cs complex with *M*Cl_2_ (*M* = Pt, Pd) by Kumar *et al.* (2017 ▸). Notably, such compounds predominantly constitute unanti­cipated side products and the focus of the respective characterization lies in crystallographic analyses with respect to solid-state inter­molecular inter­actions and packing motifs. More in-depth studies have focused predominatly on their inter­esting redox properties (Breitzer *et al.*, 2001 ▸; Matsubayashi *et al.*, 2002 ▸).

Only two analogous diselenide compounds with Se—Se moieties linking two 1,3-ene-di­thiol-2-*thione* moieties are reported in the literature, albeit without crystallographic data (Cerrada *et al.*, 1999 ▸; Takimiya *et al.*, 2002 ▸). To date, no such compounds are known with 1,3-ene-di­thiol-2-*one* moieties*.* A few examples are available for distantly related compounds in which cyclic alkenes are bridged by a diselenide moiety. Already in 1981, the synthesis, characterization and crystal structure of such a diselenide was described by Ruban *et al.*: bis­{4-(2-thien­yl)selenolo[3,4-*b*]thio­phen-6-yl}diselenide was formed unexpectedly by the reaction of 2-[(tri­phenyl­phospho­nio)meth­yl] thio­phene chloride with sodium hydrogen selenite (Ruban *et al.*, 1981 ▸). In 2000, Oilunkaniemi *et al.* published a procedure for the synthesis of thienyl- and furyl diselenide compounds, which was confirmed by respective crystal structures and selenium NMR spectra (Oilunkaniemi *et al.*, 2000 ▸). Kumar & Nangia (2000 ▸) published the crystal structure of 2,2‘-di­seleno­bis­(4,4-di­phenyl­cyclo-hexa-2,5-dienone). In 2003, Thaler *et al.* synthesized cyclo­penta­dienyl selenium compounds as multifunctional ligand systems with a varied number of selenium atoms in the Se_*n*_ bridge (Thaler *et al.*, 2003 ▸). Recently, the formation of a diselenide as a byproduct during the synthesis of heliannuol C (as confirmed by X-ray diffraction) was described by Biswas *et al.* (2017 ▸). The crystal structures of bis­[4-methyl-1,3-di­thiol-2-one] di­sulfide and diselenide described in the current work are the first in which two 1,3-ene-di­thiol-2-one moieties are linked by an S—S and an Se—Se bridge, respectively. For the latter, even the chemical structure is entirely unprecedented.

## Synthesis and crystallization   

*Preparation of bis­[4-methyl-1,3-di­thiol-2-one] di­sulfide:* This was undertaken by a modification of a published procedure (Dinsmore *et al.*, 1998 ▸). 4-Methyl-1,3-di­thiol-2-one (0.95 g, 7.2 mmol) and tri­butyl­tin chloride (2.92 ml, 8.63 mmol) in dry THF (10 ml) under nitro­gen were cooled to 169 K (N_2_/MeOH:Et_2_O or dry ice/Et_2_O), and LDA (9.8 ml, 7.9 mmol, 10% solution in hexa­ne) was added dropwise over 5 min. The mixture was allowed to stand for 35 min, warmed to ice-bath temperature and after a further 10 minutes quenched with a saturated aqueous solution of NH_4_Cl (around 20 ml). The organic phase was diluted with EtOAc, separated and the aqueous phase re-extracted with Et_2_O (2 × 15 ml). The combined organic phases were washed with brine, dried and the solvent evaporated *in vacuo* to give a yellowish oil as crude product. This was purified by chromatography (silica gel), eluting with EtOAc/petroleum ether (40/60) 3:97 *v*/*v* to give 4-methyl-5-tri-*n*-butyl­stannyl-1,3-di­thiol-2-one as the major product. During purification, a yellowish oily fraction was isolated and subsequently stored at 253 K, forming large yellow crystals. Crystallographic evaluation of these crystals reveals the formation of the side product bis­[4-methyl-1,3-di­thiol-2-one] di­sulfide.

*Preparation of bis­[4-methyl-1,3-di­thiol-2-one] diselenide:* The synthesis was carried out under an inert gas atmosphere of nitro­gen, whereas the purification steps were carried out in air. To a solution of 4-methyl-5-tri-*n*-butyl­stannyl-1,3-di­thiol-2-one (352.5 mg, 0.84 mmol) in freshly distilled dioxane (5 ml) was added freshly sublimed selenium dioxide (134.2 mg, 1.21 mmol). The reaction mixture was heated at reflux temperature for 6 h. After cooling, the solution was filtered through celite. Solvent removal gave an orange solid (188.0 mg, 0.38 mmol, 45%). Yellow crystals suitable for crystallographic analysis were obtained by recrystallization from acetone.

## Refinement   

Crystal data, data collection and structure refinement details are summarized in Table 3 ▸. The six methyl hydrogen atoms of each structure were included in calculated positions and treated as riding with C—H = 0.98 Å and *U_i_*_so_(H) = 1.5*U*_eq_(C).

## Supplementary Material

Crystal structure: contains datablock(s) CSV72a12, it14ii. DOI: 10.1107/S2056989018007454/wm5446sup1.cif

Structure factors: contains datablock(s) CSV72a12. DOI: 10.1107/S2056989018007454/wm5446CSV72a12sup2.hkl

Structure factors: contains datablock(s) it14ii. DOI: 10.1107/S2056989018007454/wm5446it14iisup3.hkl

CCDC references: 1843766, 1843765

Additional supporting information:  crystallographic information; 3D view; checkCIF report

## Figures and Tables

**Figure 2:**
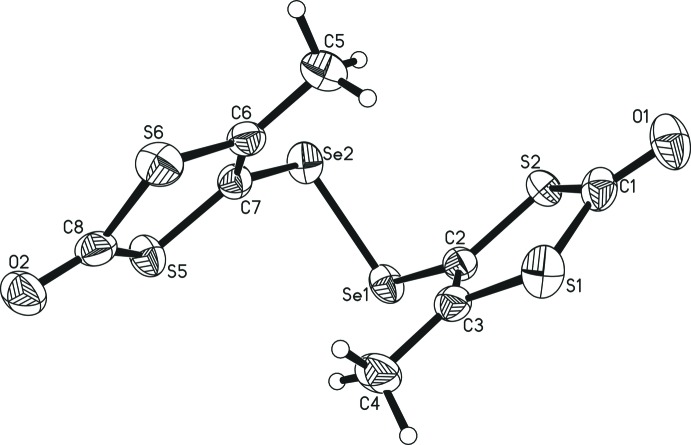
The mol­ecular structure of [bis­[4-methyl-1,3-ene-di­thiol-2-one] diselenide. Displacement ellipsoids are shown at the 50% probability level.

**Table 1:** Hydrogen-bond geometry (Å, °) for C_8_H_6_O_2_S_6_ [Chem scheme1]

*D*—H⋯*A*	*D*—H	H⋯*A*	*D*⋯*A*	*D*—H⋯*A*
C4—H4*A*⋯S3	0.98	2.76	3.244 (2)	111
C5—H5*A*⋯S4	0.98	2.75	3.234 (2)	111
C4—H4*B*⋯O1^i^	0.98	2.53	3.345 (2)	141
C5—H5*C*⋯S3^ii^	0.98	3.14	3.8063 (19)	126
C5—H5*C*⋯S5^iii^	0.98	3.01	3.825 (2)	142
C4—H4*C*⋯S2^iv^	0.98	3.12	4.021 (2)	153

Symmetry codes: (i) 
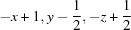
; (ii) 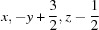; (iii) 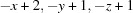; (iv) 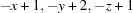.

**Table 2:** Hydrogen-bond geometry (Å, °) for C_8_H_6_O_2_S_4_Se_2_ [Chem scheme1]

*D*—H⋯*A*	*D*—H	H⋯*A*	*D*⋯*A*	*D*—H⋯*A*
C4—H4*A*⋯Se1	0.98	2.84	3.354 (4)	114
C5—H5*A*⋯Se2	0.98	2.83	3.341 (4)	114
C4—H4*B*⋯O1^i^	0.98	2.55	3.369 (5)	141
C5—H5*C*⋯Se1^ii^	0.98	3.14	3.801 (4)	126
C5—H5*C*⋯S5^iii^	0.98	3.04	3.850 (4)	141
C4—H4*C*⋯S2^iv^	0.98	3.13	3.992 (4)	148

Symmetry codes: (i) 
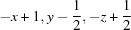
; (ii) 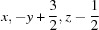; (iii) 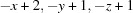; (iv) 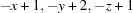.

**Table 3:** Experimental details

	C_8_H_6_O_2_S_6_	C_8_H_6_O_2_S_4_Se_2_
Crystal data		
*M*_r_	326.49	420.29
Crystal system, space group	Monoclinic, *P*2_1_/*c*	Monoclinic, *P*2_1_/*c*
Temperature (K)	170	170
*a*, *b*, *c* (Å)	10.845 (2), 9.0387 (18), 13.370 (3)	10.960 (2), 9.1348 (18), 13.495 (3)
β (°)	108.95 (3)	108.29 (3)
*V* (Å^3^)	1239.6 (4)	1282.8 (5)
*Z*	4	4
Radiation type	Mo *K*α	Mo *K*α
μ (mm^−1^)	1.08	6.40
Crystal size (mm)	0.50 × 0.20 × 0.001	0.48 × 0.43 × 0.41

Data collection		
Diffractometer	STOE IPDS2T	Stoe IPDS2T
Absorption correction	Numerical face indexed	Numerical face indexed
*T*_min_, *T*_max_	0.771, 0.942	0.393, 0.786
No. of measured, independent and observed [*I* > 2σ(*I*)] reflections	13324, 3344, 2636	10805, 2733, 2009
*R*_int_	0.034	0.063
(sin θ/λ)_max_ (Å^−1^)	0.687	0.636

Refinement		
*R*[*F* ^2^ > 2σ(*F* ^2^)], *wR*(*F* ^2^), *S*	0.027, 0.064, 1.03	0.029, 0.058, 0.97
No. of reflections	3344	2733
No. of parameters	147	147
H-atom treatment	H-atom parameters constrained	H-atom parameters constrained
Δρ_max_, Δρ_min_ (e Å^−3^)	0.36, −0.34	0.41, −0.51

Computer programs: *X-AREA* (Stoe & Cie, 2010 ▸), *SIR92* (Altomare *et al.*, 1994 ▸), *SHELXS2016/6* (Sheldrick, 2008 ▸), *SHELXL2016/6* (Sheldrick, 2015 ▸), *XP* (Bruker, 1998 ▸), *DIAMOND* (Brandenburg, 2001 ▸), *Mercury* (Macrae *et al.*, 2006 ▸) and *CIFTAB* (Sheldrick, 2015 ▸).

## supplementary crystallographic information

### 4,4'-(Disulfane-1,2-diyl)bis(5-methyl-2H-1,3-dithiol-2-one) (CSV72a12) Crystal data

**Table:**

C_8_H_6_O_2_S_4_Se_2_	*F*(000) = 808
*M**_r_* = 420.29	*D*_x_ = 2.176 Mg m^−^^3^
Monoclinic, *P*2_1_/*c*	Mo *K*α radiation, λ = 0.71073 Å
*a* = 10.960 (2) Å	Cell parameters from 11124 reflections
*b* = 9.1348 (18) Å	θ = 3.2–53.8°
*c* = 13.495 (3) Å	µ = 6.40 mm^−^^1^
β = 108.29 (3)°	*T* = 170 K
*V* = 1282.8 (5) Å^3^	Block, yellow
*Z* = 4	0.48 × 0.43 × 0.41 mm

### 4,4'-(Disulfane-1,2-diyl)bis(5-methyl-2H-1,3-dithiol-2-one) (CSV72a12) Data collection

**Table:**

Stoe IPDS2T diffractometer	2733 independent reflections
Radiation source: fine-focus sealed tube	2009 reflections with *I* > 2σ(*I*)
Detector resolution: 6.67 pixels mm^-1^	*R*_int_ = 0.063
ω scans	θ_max_ = 26.9°, θ_min_ = 2.0°
Absorption correction: numerical face indexed	*h* = −13→13
*T*_min_ = 0.393, *T*_max_ = 0.786	*k* = −11→11
10805 measured reflections	*l* = −17→17

### 4,4'-(Disulfane-1,2-diyl)bis(5-methyl-2H-1,3-dithiol-2-one) (CSV72a12) Refinement

**Table:**

Refinement on *F*^2^	0 restraints
Least-squares matrix: full	Hydrogen site location: inferred from neighbouring sites
*R*[*F*^2^ > 2σ(*F*^2^)] = 0.029	H-atom parameters constrained
*wR*(*F*^2^) = 0.058	*w* = 1/[σ^2^(*F*_o_^2^) + (0.0249*P*)^2^] where *P* = (*F*_o_^2^ + 2*F*_c_^2^)/3
*S* = 0.97	(Δ/σ)_max_ = 0.001
2733 reflections	Δρ_max_ = 0.41 e Å^−^^3^
147 parameters	Δρ_min_ = −0.51 e Å^−^^3^

### 4,4'-(Disulfane-1,2-diyl)bis(5-methyl-2H-1,3-dithiol-2-one) (CSV72a12) Special details

**Table:**

Experimental. (*X-RED32* and *X-SHAPE*; Stoe, 2010)
Geometry. All esds (except the esd in the dihedral angle between two l.s. planes) are estimated using the full covariance matrix. The cell esds are taken into account individually in the estimation of esds in distances, angles and torsion angles; correlations between esds in cell parameters are only used when they are defined by crystal symmetry. An approximate (isotropic) treatment of cell esds is used for estimating esds involving l.s. planes.

### 4,4'-(Disulfane-1,2-diyl)bis(5-methyl-2H-1,3-dithiol-2-one) (CSV72a12) Fractional atomic coordinates and isotropic or equivalent isotropic displacement parameters (Å^2^)

**Table:**

	*x*	*y*	*z*	*U*_iso_*/*U*_eq_	
C1	0.5603 (4)	0.9723 (4)	0.2588 (3)	0.0330 (8)	
C2	0.6767 (3)	0.9491 (4)	0.4583 (3)	0.0283 (7)	
C3	0.5772 (3)	0.8578 (4)	0.4418 (3)	0.0292 (7)	
C4	0.5451 (4)	0.7610 (4)	0.5199 (3)	0.0407 (9)	
H4A	0.610175	0.773264	0.588334	0.061*	
H4B	0.543631	0.658702	0.497773	0.061*	
H4C	0.460552	0.787918	0.524643	0.061*	
C5	0.8143 (4)	0.6153 (5)	0.3661 (3)	0.0430 (10)	
H5A	0.857954	0.708541	0.364796	0.064*	
H5B	0.722081	0.626480	0.330068	0.064*	
H5C	0.849415	0.539960	0.331030	0.064*	
C6	0.8349 (3)	0.5707 (4)	0.4771 (3)	0.0300 (7)	
C7	0.8914 (3)	0.6483 (4)	0.5642 (3)	0.0288 (7)	
C8	0.8162 (4)	0.4074 (4)	0.6323 (3)	0.0333 (8)	
O1	0.5276 (3)	1.0021 (3)	0.16730 (19)	0.0441 (7)	
O2	0.7920 (3)	0.3109 (3)	0.6837 (2)	0.0445 (7)	
S1	0.47447 (9)	0.85051 (10)	0.31380 (8)	0.0387 (2)	
S2	0.69642 (9)	1.04502 (9)	0.35303 (6)	0.02911 (19)	
S5	0.89572 (10)	0.57233 (10)	0.68357 (7)	0.0345 (2)	
S6	0.77748 (10)	0.39942 (10)	0.49537 (7)	0.0368 (2)	
Se1	0.80328 (4)	0.98085 (4)	0.58945 (3)	0.03435 (11)	
Se2	0.96657 (4)	0.83504 (4)	0.56427 (3)	0.03646 (11)	

### 4,4'-(Disulfane-1,2-diyl)bis(5-methyl-2H-1,3-dithiol-2-one) (CSV72a12) Atomic displacement parameters (Å^2^)

**Table:**

	*U*^11^	*U*^22^	*U*^33^	*U*^12^	*U*^13^	*U*^23^
C1	0.039 (2)	0.0238 (17)	0.0322 (19)	0.0044 (15)	0.0063 (16)	−0.0015 (14)
C2	0.0338 (19)	0.0256 (16)	0.0271 (17)	0.0018 (14)	0.0116 (15)	−0.0010 (13)
C3	0.0321 (19)	0.0247 (17)	0.0331 (18)	0.0017 (14)	0.0135 (16)	−0.0005 (13)
C4	0.045 (2)	0.036 (2)	0.048 (2)	−0.0067 (17)	0.026 (2)	0.0043 (17)
C5	0.050 (3)	0.049 (2)	0.032 (2)	0.0010 (19)	0.0172 (19)	−0.0009 (16)
C6	0.0266 (18)	0.0348 (18)	0.0300 (18)	0.0022 (15)	0.0108 (15)	−0.0002 (14)
C7	0.0258 (18)	0.0295 (17)	0.0308 (18)	0.0004 (14)	0.0085 (15)	0.0014 (14)
C8	0.037 (2)	0.0308 (18)	0.0375 (19)	0.0024 (16)	0.0188 (17)	−0.0044 (16)
O1	0.0597 (18)	0.0337 (14)	0.0289 (13)	−0.0022 (13)	−0.0007 (13)	−0.0016 (11)
O2	0.0629 (19)	0.0320 (14)	0.0475 (15)	−0.0064 (13)	0.0303 (15)	−0.0005 (12)
S1	0.0328 (5)	0.0319 (5)	0.0450 (5)	−0.0072 (4)	0.0030 (4)	0.0015 (4)
S2	0.0334 (5)	0.0295 (4)	0.0240 (4)	−0.0052 (4)	0.0082 (4)	−0.0003 (3)
S5	0.0448 (6)	0.0306 (4)	0.0256 (4)	−0.0044 (4)	0.0075 (4)	−0.0008 (3)
S6	0.0432 (6)	0.0337 (5)	0.0331 (5)	−0.0064 (4)	0.0116 (4)	−0.0095 (4)
Se1	0.0484 (2)	0.02951 (19)	0.02304 (17)	−0.00266 (16)	0.00823 (15)	−0.00313 (14)
Se2	0.0296 (2)	0.0350 (2)	0.0410 (2)	−0.00724 (16)	0.00564 (16)	0.00402 (16)

### 4,4'-(Disulfane-1,2-diyl)bis(5-methyl-2H-1,3-dithiol-2-one) (CSV72a12) Geometric parameters (Å, º)

**Table:**

C1—O1	1.203 (4)	C5—H5A	0.9800
C1—S2	1.759 (4)	C5—H5B	0.9800
C1—S1	1.765 (4)	C5—H5C	0.9800
C2—C3	1.335 (5)	C6—C7	1.346 (5)
C2—S2	1.739 (3)	C6—S6	1.733 (4)
C2—Se1	1.898 (4)	C7—S5	1.741 (3)
C3—C4	1.499 (5)	C7—Se2	1.895 (3)
C3—S1	1.742 (4)	C8—O2	1.202 (4)
C4—H4A	0.9800	C8—S6	1.763 (4)
C4—H4B	0.9800	C8—S5	1.769 (4)
C4—H4C	0.9800	Se1—Se2	2.3397 (7)
C5—C6	1.498 (5)		
			
O1—C1—S2	124.6 (3)	C6—C5—H5C	109.5
O1—C1—S1	123.2 (3)	H5A—C5—H5C	109.5
S2—C1—S1	112.15 (19)	H5B—C5—H5C	109.5
C3—C2—S2	118.9 (3)	C7—C6—C5	127.9 (3)
C3—C2—Se1	124.8 (3)	C7—C6—S6	116.0 (3)
S2—C2—Se1	116.29 (19)	C5—C6—S6	116.1 (3)
C2—C3—C4	127.6 (3)	C6—C7—S5	118.1 (3)
C2—C3—S1	115.4 (3)	C6—C7—Se2	123.7 (3)
C4—C3—S1	117.0 (3)	S5—C7—Se2	118.23 (18)
C3—C4—H4A	109.5	O2—C8—S6	123.4 (3)
C3—C4—H4B	109.5	O2—C8—S5	124.7 (3)
H4A—C4—H4B	109.5	S6—C8—S5	111.8 (2)
C3—C4—H4C	109.5	C3—S1—C1	97.42 (17)
H4A—C4—H4C	109.5	C2—S2—C1	96.07 (17)
H4B—C4—H4C	109.5	C7—S5—C8	96.36 (17)
C6—C5—H5A	109.5	C6—S6—C8	97.68 (17)
C6—C5—H5B	109.5	C7—Se2—Se1	99.23 (10)
H5A—C5—H5B	109.5	C2—Se1—Se2	98.40 (10)

### 4,4'-(Disulfane-1,2-diyl)bis(5-methyl-2H-1,3-dithiol-2-one) (CSV72a12) Hydrogen-bond geometry (Å, º)

**Table:**

*D*—H···*A*	*D*—H	H···*A*	*D*···*A*	*D*—H···*A*
C4—H4*A*···Se1	0.98	2.84	3.354 (4)	114
C5—H5*A*···Se2	0.98	2.83	3.341 (4)	114
C4—H4*B*···O1^i^	0.98	2.55	3.369 (5)	141
C5—H5*C*···Se1^ii^	0.98	3.14	3.801 (4)	126
C5—H5*C*···S5^iii^	0.98	3.04	3.850 (4)	141
C4—H4*C*···S2^iv^	0.98	3.13	3.992 (4)	148

Symmetry codes: (i) −*x*+1, *y*−1/2, −*z*+1/2; (ii) *x*, −*y*+3/2, *z*−1/2; (iii) −*x*+2, −*y*+1, −*z*+1; (iv) −*x*+1, −*y*+2, −*z*+1.

### 4,4'-(Diselanane-1,2-diyl)bis(5-methyl-2H-1,3-dithiol-2-one) (it14ii) Crystal data

**Table:**

C_8_H_6_O_2_S_6_	*F*(000) = 664
*M**_r_* = 326.49	*D*_x_ = 1.749 Mg m^−^^3^
Monoclinic, *P*2_1_/*c*	Mo *K*α radiation, λ = 0.71073 Å
Hall symbol: -P 2ybc	Cell parameters from 13753 reflections
*a* = 10.845 (2) Å	θ = 6.4–58.5°
*b* = 9.0387 (18) Å	µ = 1.08 mm^−^^1^
*c* = 13.370 (3) Å	*T* = 170 K
β = 108.95 (3)°	Plate, yellow
*V* = 1239.6 (4) Å^3^	0.50 × 0.20 × 0.001 mm
*Z* = 4	

### 4,4'-(Diselanane-1,2-diyl)bis(5-methyl-2H-1,3-dithiol-2-one) (it14ii) Data collection

**Table:**

STOE IPDS2T diffractometer	3344 independent reflections
Radiation source: fine-focus sealed tube	2636 reflections with *I* > 2σ(*I*)
Detector resolution: 6.67 pixels mm^-1^	*R*_int_ = 0.034
ω scans	θ_max_ = 29.2°, θ_min_ = 3.2°
Absorption correction: numerical face indexed	*h* = −14→11
*T*_min_ = 0.771, *T*_max_ = 0.942	*k* = −12→12
13324 measured reflections	*l* = −18→18

### 4,4'-(Diselanane-1,2-diyl)bis(5-methyl-2H-1,3-dithiol-2-one) (it14ii) Refinement

**Table:**

Refinement on *F*^2^	Primary atom site location: structure-invariant direct methods
Least-squares matrix: full	Secondary atom site location: difference Fourier map
*R*[*F*^2^ > 2σ(*F*^2^)] = 0.027	Hydrogen site location: inferred from neighbouring sites
*wR*(*F*^2^) = 0.064	H-atom parameters constrained
*S* = 1.03	*w* = 1/[σ^2^(*F*_o_^2^) + (0.031*P*)^2^ + 0.2272*P*] where *P* = (*F*_o_^2^ + 2*F*_c_^2^)/3
3344 reflections	(Δ/σ)_max_ = 0.001
147 parameters	Δρ_max_ = 0.36 e Å^−^^3^
0 restraints	Δρ_min_ = −0.34 e Å^−^^3^

### 4,4'-(Diselanane-1,2-diyl)bis(5-methyl-2H-1,3-dithiol-2-one) (it14ii) Special details

**Table:**

Experimental. (*X-RED32* and *X-SHAPE*; Stoe, 2010)
Geometry. All esds (except the esd in the dihedral angle between two l.s. planes) are estimated using the full covariance matrix. The cell esds are taken into account individually in the estimation of esds in distances, angles and torsion angles; correlations between esds in cell parameters are only used when they are defined by crystal symmetry. An approximate (isotropic) treatment of cell esds is used for estimating esds involving l.s. planes.
Refinement. Refinement of F^2^ against ALL reflections. The weighted R-factor wR and goodness of fit S are based on F^2^, conventional R-factors R are based on F, with F set to zero for negative F^2^. The threshold expression of F^2^ > 2σ(F^2^) is used only for calculating R-factors(gt) etc. and is not relevant to the choice of reflections for refinement. R-factors based on F^2^ are statistically about twice as large as those based on F, and R- factors based on ALL data will be even larger.

### 4,4'-(Diselanane-1,2-diyl)bis(5-methyl-2H-1,3-dithiol-2-one) (it14ii) Fractional atomic coordinates and isotropic or equivalent isotropic displacement parameters (Å^2^)

**Table:**

	*x*	*y*	*z*	*U*_iso_*/*U*_eq_	
C1	0.56140 (18)	0.97691 (18)	0.25987 (13)	0.0303 (3)	
C2	0.68624 (16)	0.94540 (18)	0.46161 (11)	0.0264 (3)	
C3	0.58060 (16)	0.85986 (19)	0.44516 (12)	0.0283 (3)	
C4	0.54816 (19)	0.7632 (2)	0.52398 (15)	0.0377 (4)	
H4A	0.616846	0.771796	0.592700	0.057*	
H4B	0.541661	0.660179	0.500052	0.057*	
H4C	0.464737	0.794436	0.530838	0.057*	
C5	0.8123 (2)	0.6221 (2)	0.36279 (14)	0.0432 (4)	
H5A	0.856072	0.716664	0.361746	0.065*	
H5B	0.718458	0.633249	0.326446	0.065*	
H5C	0.847281	0.546622	0.326768	0.065*	
C6	0.83515 (16)	0.5762 (2)	0.47454 (13)	0.0310 (3)	
C7	0.89133 (16)	0.6551 (2)	0.56261 (13)	0.0298 (3)	
C8	0.82334 (18)	0.4069 (2)	0.63253 (14)	0.0345 (4)	
O1	0.52663 (15)	1.00821 (15)	0.16765 (9)	0.0419 (3)	
O2	0.80246 (16)	0.30792 (16)	0.68404 (11)	0.0476 (3)	
S1	0.47149 (4)	0.85968 (5)	0.31651 (4)	0.03579 (11)	
S2	0.70545 (4)	1.04272 (5)	0.35471 (3)	0.02809 (9)	
S3	0.80927 (5)	0.96538 (5)	0.58353 (3)	0.03365 (10)	
S4	0.95479 (4)	0.83296 (5)	0.56233 (4)	0.03649 (11)	
S5	0.89988 (5)	0.57621 (5)	0.68428 (3)	0.03564 (11)	
S6	0.78190 (5)	0.40033 (5)	0.49318 (3)	0.03672 (11)	

### 4,4'-(Diselanane-1,2-diyl)bis(5-methyl-2H-1,3-dithiol-2-one) (it14ii) Atomic displacement parameters (Å^2^)

**Table:**

	*U*^11^	*U*^22^	*U*^33^	*U*^12^	*U*^13^	*U*^23^
C1	0.0363 (9)	0.0215 (8)	0.0301 (7)	0.0009 (6)	0.0065 (6)	−0.0033 (6)
C2	0.0309 (8)	0.0246 (8)	0.0253 (7)	0.0014 (6)	0.0113 (6)	−0.0017 (6)
C3	0.0306 (8)	0.0246 (8)	0.0321 (7)	0.0016 (6)	0.0136 (6)	−0.0012 (6)
C4	0.0428 (10)	0.0325 (9)	0.0453 (9)	−0.0018 (8)	0.0247 (8)	0.0031 (7)
C5	0.0495 (11)	0.0536 (12)	0.0298 (8)	0.0029 (9)	0.0174 (8)	−0.0026 (8)
C6	0.0253 (8)	0.0377 (9)	0.0321 (7)	0.0022 (7)	0.0125 (6)	−0.0032 (7)
C7	0.0232 (7)	0.0338 (9)	0.0313 (7)	0.0011 (6)	0.0075 (6)	−0.0001 (6)
C8	0.0353 (9)	0.0333 (9)	0.0383 (8)	0.0016 (7)	0.0164 (7)	−0.0036 (7)
O1	0.0567 (9)	0.0324 (7)	0.0277 (6)	−0.0020 (6)	0.0014 (6)	−0.0005 (5)
O2	0.0646 (10)	0.0338 (7)	0.0514 (8)	−0.0031 (7)	0.0285 (7)	0.0010 (6)
S1	0.0296 (2)	0.0294 (2)	0.0422 (2)	−0.00548 (17)	0.00317 (17)	0.00138 (17)
S2	0.0311 (2)	0.0284 (2)	0.02544 (17)	−0.00528 (16)	0.01009 (15)	−0.00122 (14)
S3	0.0417 (2)	0.0317 (2)	0.02435 (17)	−0.00303 (18)	0.00641 (16)	−0.00476 (15)
S4	0.0255 (2)	0.0372 (2)	0.0426 (2)	−0.00639 (17)	0.00532 (17)	0.00179 (18)
S5	0.0422 (2)	0.0332 (2)	0.02811 (18)	−0.00173 (18)	0.00668 (16)	−0.00185 (16)
S6	0.0386 (2)	0.0352 (2)	0.0367 (2)	−0.00491 (18)	0.01269 (18)	−0.01011 (17)

### 4,4'-(Diselanane-1,2-diyl)bis(5-methyl-2H-1,3-dithiol-2-one) (it14ii) Geometric parameters (Å, º)

**Table:**

C1—O1	1.200 (2)	C5—H5A	0.9800
C1—S2	1.7652 (19)	C5—H5B	0.9800
C1—S1	1.7682 (19)	C5—H5C	0.9800
C2—C3	1.340 (2)	C6—C7	1.342 (2)
C2—S2	1.7473 (16)	C6—S6	1.7363 (19)
C2—S3	1.7486 (17)	C7—S4	1.7494 (18)
C3—C4	1.496 (2)	C7—S5	1.7507 (17)
C3—S1	1.7426 (18)	C8—O2	1.195 (2)
C4—H4A	0.9800	C8—S6	1.7700 (18)
C4—H4B	0.9800	C8—S5	1.7710 (19)
C4—H4C	0.9800	S3—S4	2.0723 (7)
C5—C6	1.492 (2)		
			
O1—C1—S2	124.61 (15)	C6—C5—H5C	109.5
O1—C1—S1	123.25 (15)	H5A—C5—H5C	109.5
S2—C1—S1	112.13 (9)	H5B—C5—H5C	109.5
C3—C2—S2	118.62 (12)	C7—C6—C5	127.68 (18)
C3—C2—S3	124.44 (12)	C7—C6—S6	115.98 (13)
S2—C2—S3	116.93 (10)	C5—C6—S6	116.34 (14)
C2—C3—C4	127.26 (16)	C6—C7—S4	123.61 (14)
C2—C3—S1	115.65 (12)	C6—C7—S5	118.09 (14)
C4—C3—S1	117.07 (13)	S4—C7—S5	118.30 (10)
C3—C4—H4A	109.5	O2—C8—S6	123.42 (15)
C3—C4—H4B	109.5	O2—C8—S5	125.06 (15)
H4A—C4—H4B	109.5	S6—C8—S5	111.52 (10)
C3—C4—H4C	109.5	C3—S1—C1	97.47 (8)
H4A—C4—H4C	109.5	C2—S2—C1	96.03 (8)
H4B—C4—H4C	109.5	C2—S3—S4	101.38 (6)
C6—C5—H5A	109.5	C7—S4—S3	102.29 (6)
C6—C5—H5B	109.5	C7—S5—C8	96.44 (8)
H5A—C5—H5B	109.5	C6—S6—C8	97.92 (8)

### 4,4'-(Diselanane-1,2-diyl)bis(5-methyl-2H-1,3-dithiol-2-one) (it14ii) Hydrogen-bond geometry (Å, º)

**Table:**

*D*—H···*A*	*D*—H	H···*A*	*D*···*A*	*D*—H···*A*
C4—H4*A*···S3	0.98	2.76	3.244 (2)	111
C5—H5*A*···S4	0.98	2.75	3.234 (2)	111
C4—H4*B*···O1^i^	0.98	2.53	3.345 (2)	141
C5—H5*C*···S3^ii^	0.98	3.14	3.8063 (19)	126
C5—H5*C*···S5^iii^	0.98	3.01	3.825 (2)	142
C4—H4*C*···S2^iv^	0.98	3.12	4.021 (2)	153

Symmetry codes: (i) −*x*+1, *y*−1/2, −*z*+1/2; (ii) *x*, −*y*+3/2, *z*−1/2; (iii) −*x*+2, −*y*+1, −*z*+1; (iv) −*x*+1, −*y*+2, −*z*+1.
